# Effect of Laser Power on Formation and Joining Strength of DP980-CFRP Joint Fabricated by Laser Circle Welding

**DOI:** 10.3390/polym17070997

**Published:** 2025-04-07

**Authors:** Sendong Ren, Yihao Shen, Taowei Wang, Hao Chen, Ninshu Ma, Jianguo Yang

**Affiliations:** 1 College of Mechanical Engineering, Zhejiang University of Technology, Hangzhou 310014, Chinayangjg@zjut.edu.cn (J.Y.); 2 Joining and Welding Research Institute, Osaka University, Osaka 567-0047, Japan; ma.ninshu.jwri@osaka-u.ac.jp

**Keywords:** laser circle welding, DP980, CFRP, FEM, joining strength

## Abstract

In the present research, laser circle welding (LCW) was proposed to join dual-phase steel (DP980) and carbon fiber-reinforced plastic (CFRP). The welding appearance, cross-section of the welded joint and fracture surfaces were subjected to multi-scale characterizations. Joining strength was evaluated by the single-lap shear test. Moreover, a numerical model was established based on the in-house finite element (FE) code JWRIAN-Hybrid to reproduce the thermal process of LCW. The results showed that successful bonding was achieved with a laser power higher than 300 W. The largest joining strength increased to about 1353.2 N (12.2 MPa) with 450 W laser power and then decreased under higher heat input. While the welded joint always presented brittle fracture, the joining zone could be divided into a squeezed zone (SZ), molten zone (MZ) and decomposition zone (DZ). The morphology of CFRP and chemical bonding information were distinct in each subregion. The chemical reaction between the O-C=O bond on the CFRP surface and the -OH bond on the DP980 sheet provided the joining force between dissimilar materials. Additionally, the developed FE model was effective in predicting the interfacial maximum temperature distribution of LCW. The influence of laser power on the joining strength of LCW joints was dualistic in character. The joining strength variation reflected the competitive result between joining zone expansion and local bonding quality change.

## 1. Introduction

The growing requirement for lightweight design in the automobile industry has promoted the upgrading of structural materials. Alongside advanced high-strength steel (AHSS) and aluminum alloys, carbon fiber-reinforced plastic (CFRP) has been developed as a new-generation lightweight material, which presents desirable characteristics and attracts great interest from manufacturers. Compared to traditional metals, CFRP provides lower density, a higher strength-to-weight ratio and excellent thermal and corrosion resistance, as well as enormous impact absorption energy [1]. The hybrid structure of metal–CFRP dissimilar materials combines the advantages of both to achieve a balance between lightweight design and cost, which has been applied in mass-market products such as the BMW i-series [2] and Audi A8 [3]. Up to now, joining technology between dissimilar materials has been widely investigated, while it is still a significant challenge which limits the application of metal–CFRP joints.

Mechanical fastening and adhesive bonding were once the accepted joining methods to connect metal and CFRP since they can overcome the significant difference in physical properties between dissimilar materials [4]. However, the fundamental problem was the additional weight induced by fasteners and adhesives, which was contrary to the original intention of a lightweight design. Recently, some weld-based methods have been proposed to join metal and CFRP, such as laser welding [5], ultrasonic welding [6], friction welding [7] and resistance spot welding [8]. Compared to the mentioned methods, laser welding performed well in manufacturing precision, the degree of automation and welding efficiency, and relevant research was also abundant.

He et al. investigated the effect of linear heat input on the mechanical properties of DP780 and PA66 CFRP laser-welded joints [9]. The maximum tensile shear force reached about 1959.4 N at 50 J/mm. The chemical bonds of M-C and M-O were formed at the interface. Xia et al. clarified the fracture behaviors of a DP590-CFRP joint with different laser welding power [10]. Three different fracture modes were established according to the residual plastic on the steel surface. Lee et al. employed a high-power diode laser to join STS306L and CFRP [11]. The influence of pressing force on bond strength was double-edged, and a 1 N condition at a 650 °C surface temperature resulted in the best strength. Zhou et al. researched laser joining between a Ti-6V-4V titanium alloy and PEEK-CFRP [12]. Micro-arc oxidation treatment could enhance the mechanical properties of welded joints to 42.3 MPa, and the Ti-O-F and Ti-F bonds provided interfacial joining force between dissimilar materials. Li et al. studied the effect of heat input on the characteristics of a TC4-CFRTP laser-welded joint [13]. With a PA6/epoxy interlayer, the peak values of shear force could reach about 3428 N and 3876 N, respectively. The joints demonstrated three kinds of fracture modes. Ashong et al. analyzed thermal laser welding between an AZ31 Mg alloy and CFRP [14]. The H-F bond between MgF_2_ and the amide group of the polymer provided adhesion strength. Tan et al. proposed a new method to measure the interfacial temperature of AZ31B-CFRP laser welding [15]. The maximum joint shear strength was about 9 MPa with a 390 °C interface peak temperature. Wang et al. employed laser surface treatment to improve the joining strength of laser-welded CFRTP/TC4 joints [16]. The ±45° crossed groove could increase the shear force by 120% (about 3416 N). Schricker et al. discussed the thermal efficiency of the laser-assisted joining of metals and CFRPs [17]. The influence of process parameters such as sheet thickness, focal diameter, material couples and overlap conditions was clarified numerically.

As reviewed above, the laser path was preset as a straight line which crossed the overlap zone. However, a significant advantage of laser welding is its good controllability. The laser scanning path can be defined easily according to the dimensions of the overlap region in order to expand the interfacial bonding area, optimize the interface temperature distribution and improve global joining strength. Huang et al. indicated that different oscillation parameters and scanning frequencies can result in a uniform laser energy distribution on an A5083 alloy [18]. Kuzu et al. defined different laser scanning strategies and laser power levels to join SS304 and composite, with the maximum tensile force varying from 300 N to 3000 N [19]. In previous research, laser circle welding was employed to join Al5052 and CFRP [20]. The joining strength was comprehensively affected by the laser power and scanning numbers.

In the present research, laser circle welding was employed to join DP980 and PA6-CFRP. The scanning number was fixed as one circle to clarify the influence of laser power. The single-lap shear test was conducted to evaluate the mechanical performance. Multi-scale characterizations were performed on the cross-section and fracture surfaces to clarify the interfacial bonding details and fracture behaviors. Moreover, a finite element (FE) model was developed based on the in-house FE code JWRIAN-Hybrid to analyze the thermal process of laser circle welding and understand the formation of DP980-CFRP joints. The relationship between laser power, interface temperature distribution and joining strength was discussed in detail. This research made a preliminary attempt to strengthen the bearing capacity of metal–CFRP laser welded joints by changing the scanning path, which can provide a reference for the scanning strategy design in further work.

## 2. Experiment Details

### 2.1. Materials

**Figure 1:**
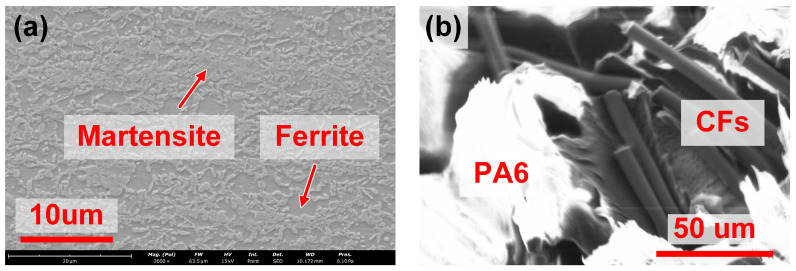
The microstructure of (**a**) DP980 and (**b**) CFRP.

In the present study, the material to be connected was HC550/980DP steel and PA6-CFRP. The chemical composition of DP980 is given in Table 1. The metal sheet was machined into an overall dimension of 75 mm×30 mm×1.5 mm. Then, the surface was polished with 1000# sandpaper to remove the oxide layer and cleaned ultrasonically in ethanol solution. The CFRP was composed of 20 wt% short-cut carbon fibers (CFs) and 80 wt% polyamide 6 (PA6, [NH-(CH_2_)_5_-CO]_n_), whose melting temperature, onset decomposition temperature and yield strength were gauged as 220 °C, 340 °C and 28.5 MPa [21], respectively. The CFRP was fabricated into 75 mm×30 mm×3 mm sheets and subjected to surface polishing to improve the roughness. The microstructures of DP980 and CFRP are depicted in Figure 1.

### 2.2. Laser Circle Welding

**Figure 2:**
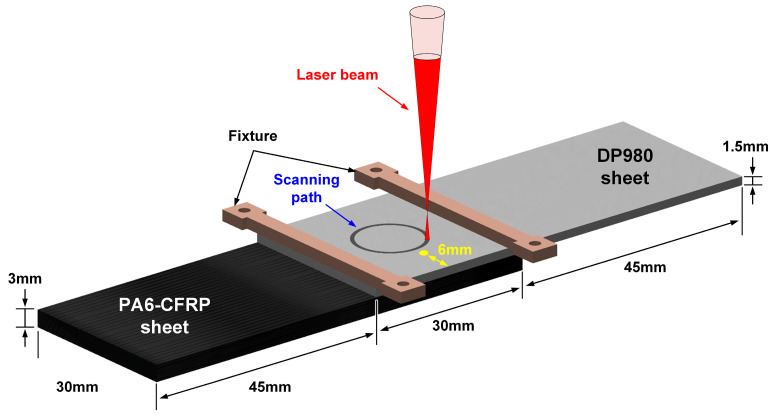
The illustration of laser circle welding between DP980 and CFRP.

The laser circle welding was processed on an HK30-Y platform swing fiber laser (Xinghong Optoelectronic Technology, Wuhan, China) with 3 kW maximum power, 1070 nm wavelength and 300 mm focal length. The welding process is illustrated in Figure 2. The DP980 sheet was lapped on the CFRP sheet with an overlap distance of 30 mm and clamped by fixtures. The laser beam was irradiated directly on the top surface of DP980 to scan a circle path in the counterclockwise direction. The duty cycle was set as 80%. Argon gas was employed to isolate the heated area from atmosphere, whose flow rate was set as 15 L/min. The welding speed and defocusing distance were defined as 12 mm/s and 93 mm, respectively. The holding time was about 300 s. Some parallel cases were assessed to clarify the effect of laser power on the formation and joining strength of laser-welded joints, and the detailed process parameters are summarized in Table 2.

During laser circle welding, a K-type thermocouple was employed to measure the thermal cycle on the upper surface of the DP980 sheet. The measurement position was 6 mm to the edge, as marked by the yellow point in Figure 2. The data were recorded by an OM-DAQ-USB-2401 device (DwyerOmega, Michigan City, IN, USA) with a 30 Hz sampling frequency.

### 2.3. Characterization Experiments

The joining strength of DP980-CFRP laser circle welded joints was evaluated via the single-lap shear (SLS) test based on an MTS E43.504 (MTS Systems, Eden Prairie, MN, USA) universal testing machine with a 20,000 N measure range. The SLS test was performed at a room temperature of 30 °C. The crosshead speed was preset as about 1 mm/min. Meanwhile, the cross-section of the welded joint was cut along the transverse direction and then subjected to cold mounting, standard sanding and polishing procedure. The DP980 was etched with 4 wt% nital solution to show the morphological microstructure. The macro-scale observation of the cross-section and fracture surfaces was performed on a Keyence VR-3200 (Keyence, Itasca, IL, USA) digital microscope. The high-magnification characterization was performed on a Phenom XL (Nanoscience Instruments, Phoenix, AZ, USA) scanning electron microscope (SEM) with an acceleration voltage of 15 kV. The energy-dispersive X-ray spectrometry (EDS) analysis was performed on an Amptek X-123 Fast SDD (Amptek, Bedford, MA, USA) to clarify the element distribution. In addition, the X-ray photoelectron spectroscopy (XPS) test was employed to analyze the chemical bond information on the fracture surface of the DP980 side, which was performed on a Thermofisher Nexsa (Thermo Fisher Scientific, Waltham, MA, USA) with Al-Kα X-ray source and 12 kV operation voltage.

## 3. Numerical Analysis

### 3.1. Finite Element Modeling

In the present research, the LCW process was analyzed numerically based on the in-house FE code JWRIAN-Hybrid, which was developed by Osaka University. The relative algorithm and detailed governing equations were introduced in the previous research [22]. The heating and cooling processes were performed with an explicit method and implicit method [23], respectively.

**Figure 3:**
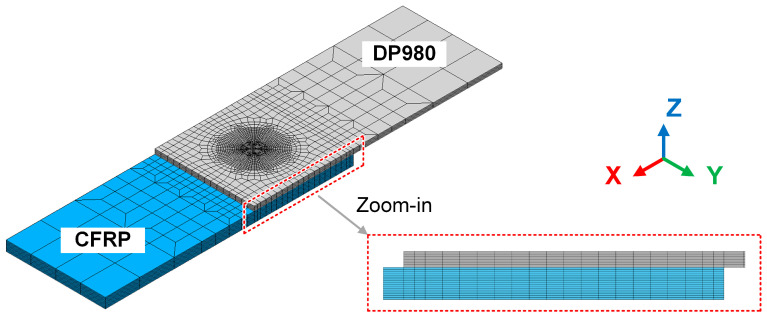
The finite element mesh of LCW.

A finite element model was established to reproduce the thermal process of LCW, as shown in Figure 3. The mesh included 40,552 structural nodes and 37,800 eight-node solid elements. The minimum element size was about 0.25 mm×0.25 mm×0.2 mm. The clamping conditions of the finite element model were identical to the actual experiment. The heat transfer coefficients with air and steel fixtures were set as $2.5\times 10^{-5}\;W\cdot {\mathrm{mm}}^{-2}\cdot K$ and $2\times 10^{-3}\;W\cdot {\mathrm{mm}}^{-2}\cdot K$, respectively.

### 3.2. Material Properties

The temperature-dependent material properties of DP980 and CFRP were necessary to ensure the accuracy of numerical simulation. The properties of CFRP were gauged experimentally in the previous study [21], and the properties of DP980 were measured via a Netzsch LFA 457 (NETZSCH-Gerätebau-GmbH, Germany) MicroFlash system, as summarized in Table 3 and Table 4, respectively.

## 4. Results and Discussion

### 4.1. Welding Appearance

**Figure 4:**
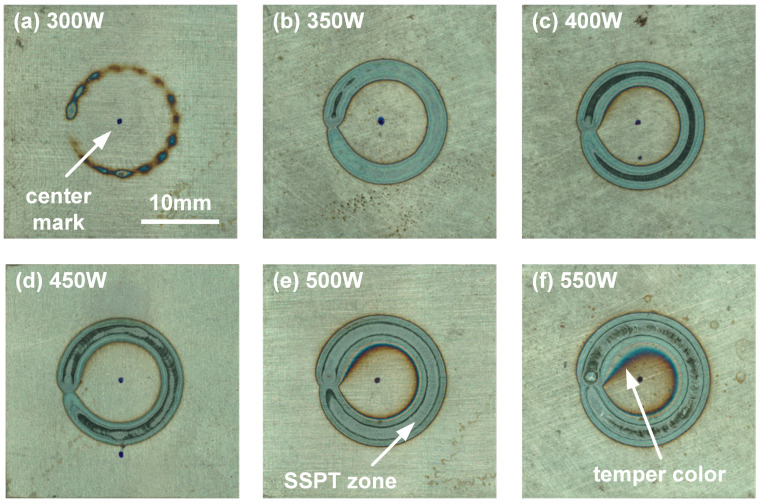
Welding appearance on the top surface of the DP980 sheet with different laser power: (**a**) 300 W, (**b**) 350 W, (**c**) 400 W, (**d**) 450 W, (**e**) 500 W and (**f**) 550 W.

The welding appearance of the laser scanning region on the DP980 sheet with different experimental conditions is compared in Figure 4. The scanning center was marked previously. In Case A, the laser power was relatively low; hence, the scanning path was not clear and was narrow and discontinuous due to the duty rate of the laser welder. When the laser power increased from 350 W to 450 W, the adequate heat input resulted in an obvious color change in the heated zone. The scanning path became dark, as seen in Figure 4c, due to the oxidation of ferrite under rapid heating and cooling. DP980 is a dual-phase steel; once the metal was heated to the Ac1 temperature (about 760 °C), solid-state phase transformation (SSPT) would occur during rapid cooling, which resulted in a different microstructure and color in the macroscopic view [24]. Therefore, some regions on scanning path were altered into a light color in Case D, and such a phenomenon was more significant in Case E, as shown in Figure 4d,e. In addition, one can find the apparent temper color of ferrite when the laser power increased to 550 W, which represented the largest heat input and surface temperature.

### 4.2. Analysis of Cross-Section

**Figure 5:**
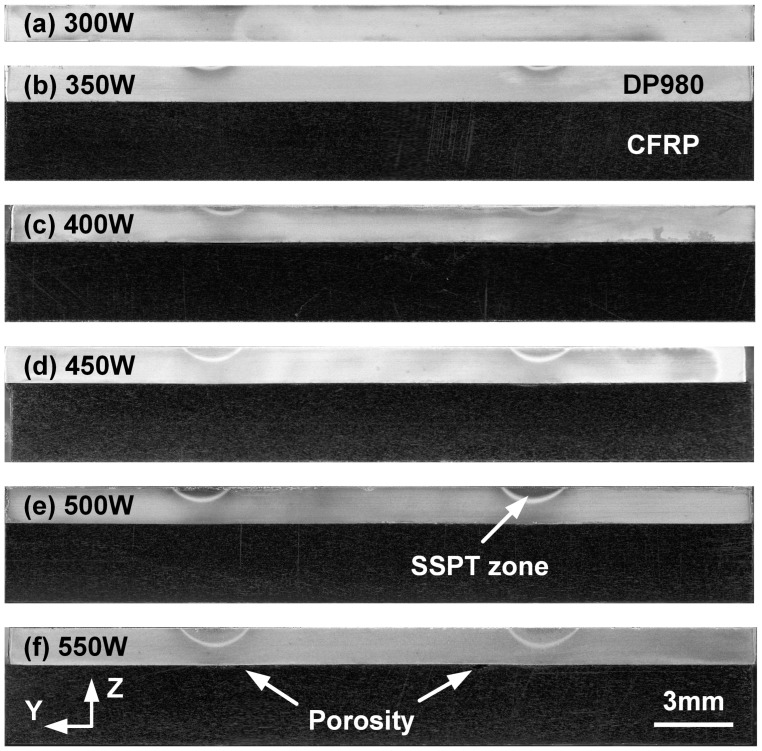
The cross-section of LCW joints with different laser power: (**a**) 300 W, (**b**) 350 W, (**c**) 400 W, (**d**) 450 W, (**e**) 500 W and (**f**) 550 W.

The cross-section of LCW joints with different welding conditions is compared in Figure 5. The light and dark sheets represented DP980 and CFRP, respectively. In Case A, the welding heat input was insufficient to achieve successful bonding. The joints were broken naturally; hence, the joining interface cannot be observed. With the increase in laser power, the joining strength was improved. In Figure 5b–f, one can observe the clear profile of the SSPT zone under the scanning path, whose dimensions grew as the laser power increased. In Case F, 550 W laser power was excessive and caused the overheating of plastics. Some porosity can be discovered on the interface below the scanning path, in which the interface temperature was highest.

**Figure 6:**
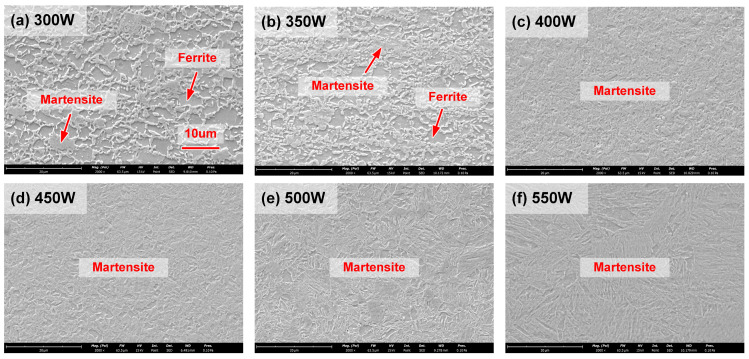
The microstructure of the SSPT zone with different laser power: (**a**) 300 W, (**b**) 350 W, (**c**) 400 W, (**d**) 450 W, (**e**) 500 W and (**f**) 550 W.

The microstructure of the SSPT zone with different welding conditions is compared in Figure 6. The heat input of Case A and Case B was not enough to influence the original phase. The microstructure was identical to the base metal, which was tempered martensite with residual ferrite, as shown in Figure 6a,b. As depicted in Figure 6c,d, the temperature was high enough to trigger the austenitization partly. Therefore, the microstructure became fine-grained martensite in Case C and Case D. With the continuous increase in laser power, the DP980 experienced complete austenitization in Case E and Case F; hence, the microstructure presented coarse-grained martensite in the SSPT zone.

**Figure 7:**
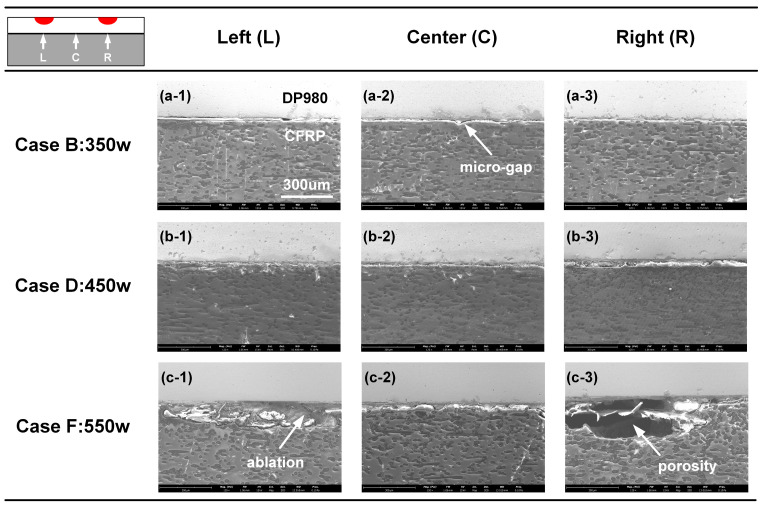
The interfacial joining status with different laser power. (**a-1**) Case B: left; (**a-2**) Case B: center; (**a-3**) Case B: right; (**b-1**) Case D: left; (**b-2**) Case D: center; (**b-3**) Case D: right; (**c-1**) Case F: left (**c-2**); Case F: center; (**c-3**) Case F: right.

The microscopic joining status on the DP980-CFRP interface with different welding conditions was analyzed via SEM, as shown in Figure 7. The left and right positions were below the 1/4 point and 3/4 point of the scanning path in the counterclockwise direction, respectively. In Case B, one can find the micro-gap at the center zone, while DP980 and CFRP were joined partly at the left and right positions, which indicated that the interface temperature in the center was lower than the outside. Overall, the 350 W laser power in Case B was insufficient to form sound bonding. When laser power was increased to 450 W in Case D, the interface temperature reached a suitable range; hence, the interfacial joining quality was optimized at the three positions, as shown in Figure 7b. Such a phenomenon represented the improved global joining strength. In Case F, the heat input was excessive and the CFRP was overheated locally. Although the interface bonding condition was good in the center, the apparent ablation of CFRP was observed on the outside. The decomposition of CFRP can generate porosity on the CFRP matrix near the interface, which was negative to the joining strength, as shown in Figure 7(c-3).

**Figure 8:**
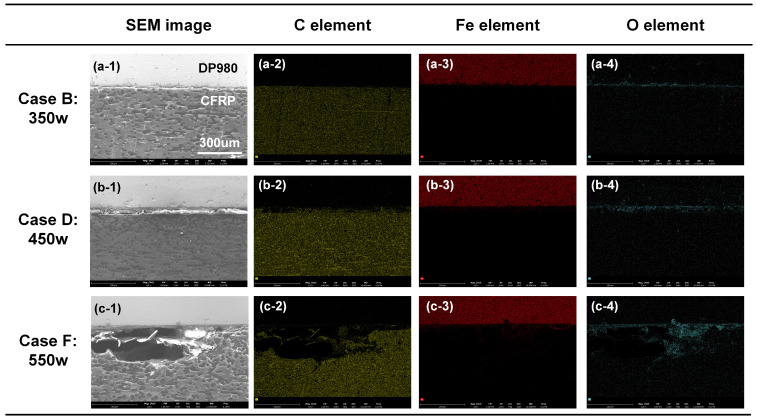
Distribution of the main elements’ distribution with different laser power. (**a-1**) Case B: SEM; (**a-2**) Case B: C element; (**a-3**) Case B: Fe element; (**a-4**) Case B: O element; (**b-1**) Case D: SEM; (**b-2**) Case D: C element; (**b-3**) Case D: Fe element; (**b-4**) Case B: O element; (**c-1**) Case F: SEM; (**c-2**) Case F: C element; (**c-3**) Case F: Fe element; (**c-4**) Case F: O element.

The EDS analysis was performed for the right position on the DP980-CFRP interface to determine the main elements’ distribution with different welding conditions, as depicted in Figure 8. In Case B, the C element and Fe element were enriched in CFRP and DP980 sheets, respectively. The signal of the O element only existed on the interface. Such an element distribution pattern was similar in Case D. The signal of the C element became weaker, accompanied by a stronger response of the O element near the interface, as shown in Figure 8b. It indicated that the CFRP tended to decompose locally. In Case F, the CFRP was decomposed significantly to generate large porosity. The signal intensity of C was relatively weak in the corresponding area. In addition, the O element was accumulated at the edge region of porosity, which suggested that the oxygen in the atmosphere joined the thermal degradation of plastic.

### 4.3. Analysis of Mechanical Performance

**Figure 9:**
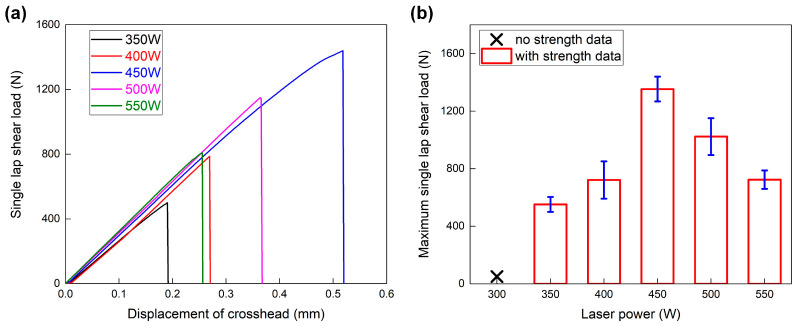
Tensile shear results of the LCW joints with different welding conditions: (**a**) typical load–displacement curves; (**b**) maximum single-lap shear load.

The mechanical performance of LCW joints was analyzed via a tensile shear test, and the load–displacement curves of different welding conditions are given in Figure 9a. The joining strength of the 300 W laser power was too weak to be fixed on the crosshead; hence, its data were lacking. Generally, the LCW joints just showed brittle fractures in all welding conditions. The load–displacement curve dropped immediately once it reached the peak, which indicated that the joining zone failed simultaneously. Meanwhile, the peak load first increased and then decreased with the continuous growth of laser power. The variation in displacement also presented an identical tendency. It indicated that the effect of laser power and the related heat input on joining strength had a dual character.

Figure 9b illustrates the relationship between laser power and maximum single-lap shear load. The successful bonding was achieved when the laser power reached 350 W, and the related joining strength was about 551.3 N. The largest joining strength appeared in Case D; the 450 W laser power resulted in a peak load of about 1353.2 N. Then, the joining strength started to degrade with higher laser power. The peak loads decreased to about 1023.2 N and 723.3 N in Case E and Case F; the relativity reduction was about 24% and 47%, respectively.

### 4.4. Analysis of Fracture Surface

**Figure 10:**
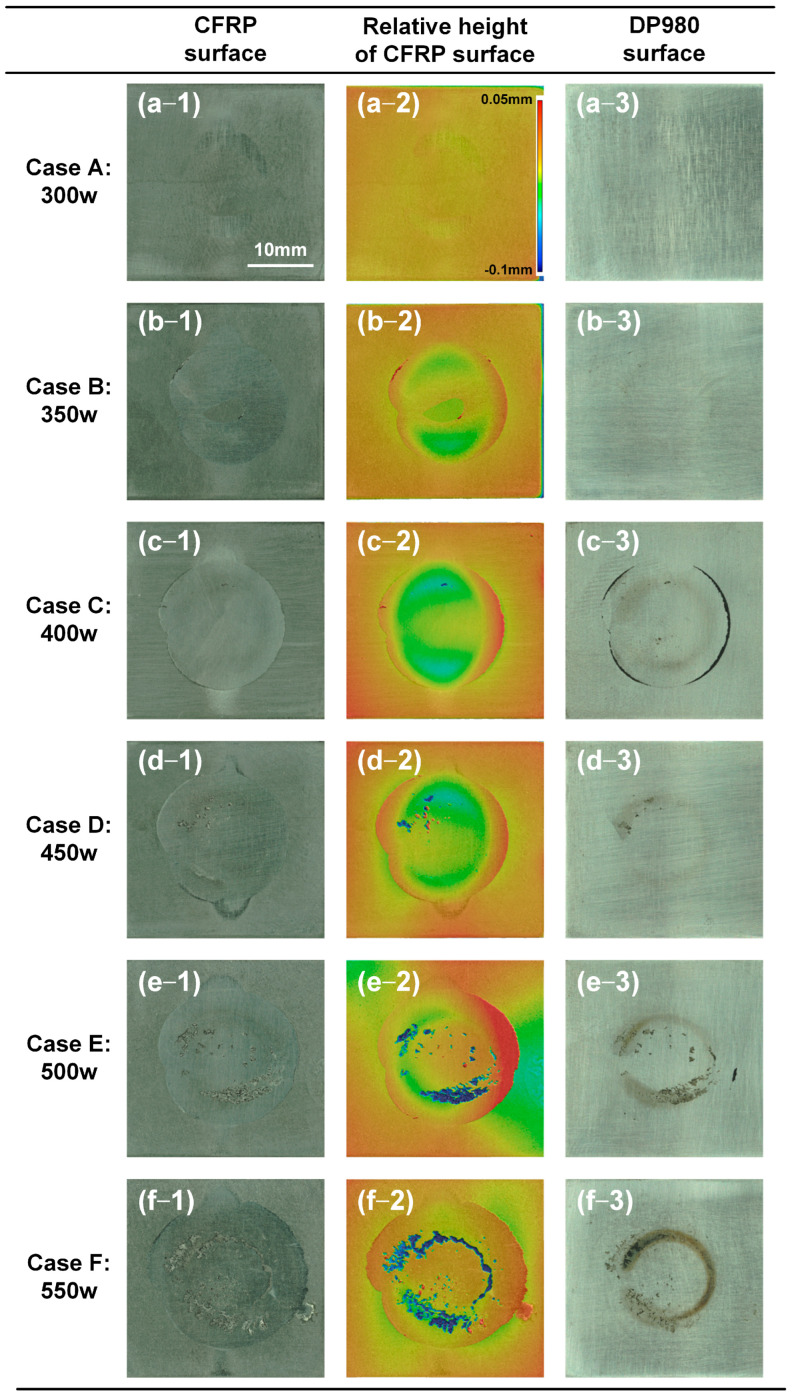
The macro-scale analysis of the fracture surface on the CFRP and DP980 sheets. (**a-1**) Case A: CFRP; (**a-2**) Case A: relative height of CFRP; (**a-3**) Case A: DP980; (**b-1**) Case B: CFRP; (**b-2**) Case B: relative height of CFRP; (**b-3**) Case B: DP980; (**c-1**) Case C: CFRP; (**c-2**) Case C: relative height of CFRP; (**c-3**) Case C: DP980; (**d-1**) Case D: CFRP; (**d-2**) Case D: relative height of CFRP; (**d-3**) Case D: DP980; (**e-1**) Case E: CFRP; (**e-2**) Case E: relative height of CFRP; (**e-3**) Case E: DP980; (**f-1**) Case F: CFRP; (**f-2**) Case F: relative height of CFRP; (**f-3**) Case F: DP980.

The macro-scale analysis of the fracture surface on CFRP and DP980 sheets was performed based on a Keyence VR-3200 digital microscope, as depicted in Figure 10. In Case A, the low laser power and heat input were insufficient; hence, the melting of plastic was unclear on the CFRP surface. The joining strength of the LCW joint was relatively weak and the surface of DP980 was clean. In Case B, the plastic below the laser scanning path was molten and expanded to both sides due to volume expansion. The joining zone presented a ring shape, and the joining strength was also improved, as shown in Figure 10(b-2). With larger laser power, more plastic was melted in Case C; therefore, the joining zone was close to a circle shape. In particular, some CFRP was residual on the DP980 sheet at the edge of the joining zone, which presented good local bonding between the plastic and DP980, as shown in Figure 10(c-3). When the laser power was higher than 450 W, one can find the apparent porosity in the joining zone, which was located below the laser scanning path due to the high interface temperature. The measured relative height was a negative value shown as a blue color, as shown in Figure 10(e-2),(f-2). Meanwhile, the decomposed and carbonated plastic was distributed on the corresponding region on the DP980 surface. The appearance of porosity can reduce the effective joining area, which leads to the degradation of joining strength.

**Figure 11:**
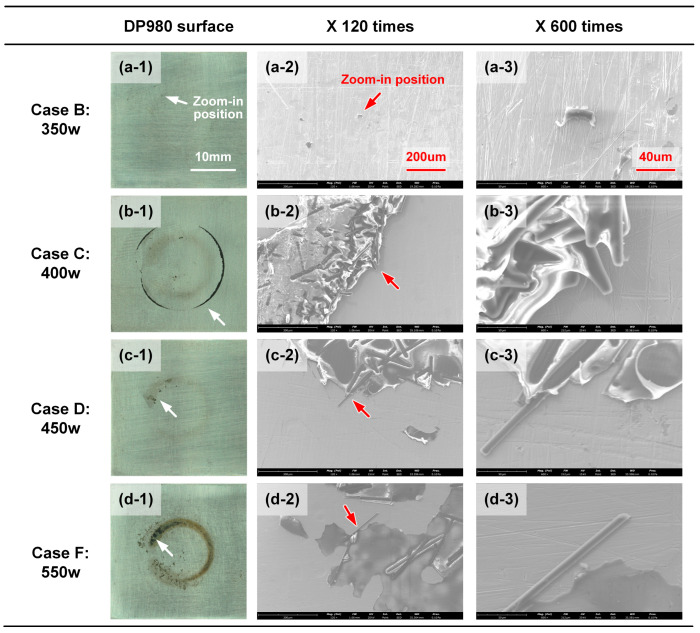
The SEM image of residual CFRP on the DP980 surface with different laser power: (**a-1**) Case B: DP980 surface; (**a-2**) Case B: X 120; (**a-3**) Case B: X 600; (**b-1**) Case C: DP980 surface; (**b-2**) Case C: X 120; (**b-3**) Case C: X 600; (**c-1**) Case D: DP980 surface; (**c-2**) Case D: X 120; (**c-3**) Case D: X 600; (**d-1**) Case F: DP980 surface; (**d-2**) Case F: X 120; (**d-3**) Case F: X 600.

The microscopic morphology of residual CFRP on the DP980 fracture surface was analyzed via SEM with 120 and 600 times magnification, as compared in Figure 11. The joining strength of Case B was relatively weak due to the low heat input. The fracture surface on the DP980 sheet was relatively clean. Only a little residual resin matrix can be observed near the end of the scanning path. In Case C, the residual CFRP was distributed at the edge of the joining zone. The carbon fibers were squeezed out partially and wrapped with much resin, as shown in Figure 11(b-3). This indicated that the molten CFRP below the scanning path was moved to both sides, which was driven by the force of thermal expansion. In Figure 11c, some lamellar CFRP remained on the DP980 surface with a brown color. In the high-magnification images, the carbon fibers were surrounded by resin matrix (light color), which depicted an apparent morphology of tearing. This indicated that the plastic experienced a significant deformation during failing, which might result in the sound bonding in Case D. The excessive heat input led to the decomposition and even carbonization of CFRP in Case F. Therefore, the lamellar CFRP with a dark color was distributed on the DP980 surface. The thickness of the residual CFRP looked quite thin and the surface was smooth, which indicated that the thermal degradation of CFRP was significant enough to cause limited local joining strength.

**Figure 12:**
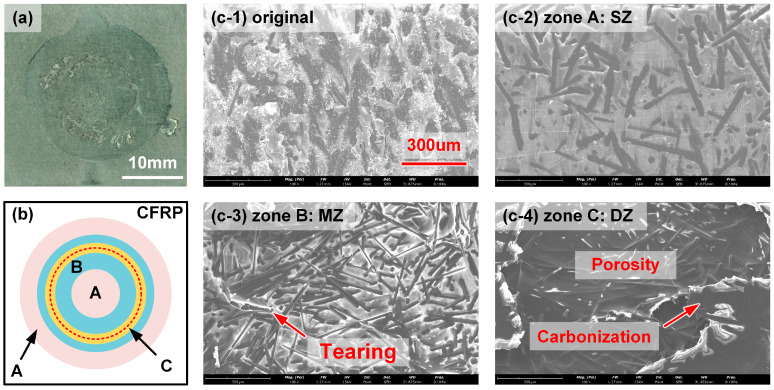
The subdivision of fracture surface according to the morphology of CFRP: (**a**) macrograph, (**b**) illustration and (**c**) SEM images of each subregion.

Case F (550 W) was employed to show the subdivision of the joining zone as an example, which is given in Figure 12. The photograph of the fracture surface on the CFRP sheet and the illustration of its components are shown in Figure 12a,b, respectively. The dashed line represented the laser scanning path. The original CFRP in the overlap zone was polished with sandpaper before welding; hence, the surface was quite rough. There were many microcracks and wrinkles on the resin matrix, as shown in Figure 12(c-1).

The joining zone included three parts. Zone A was located at the inner and outer joining zone, which was marked pink in Figure 12b. The molten plastic below the laser scanning path was squeezed here by the thermal expansion effect, which can be defined as the squeezed zone (SZ). The relative height was higher than the original surface. In addition, the interface temperature in the SZ was too low to form a strong bonding; therefore, the surface was significantly smooth, as shown in Figure 12(c-2). Zone B was close to the laser scanning path, and the interface temperature was high enough to melt the resin matrix, which can be defined as the molten zone (MZ). Although the joints were also broken along interface here, the local joining strength was better and the resin matrix experienced more deformation before failing. Therefore, many more carbon fibers were exposed on the surface, and the tearing of plastic can be discovered locally, as shown in Figure 12(c-3). It should be mentioned that the SZ and MZ were distinct in the interface temperature, while the boundary was invisible in the macroscopic observation.

If the laser power was excessive, such as in Case E and Case F, one can find that zone C occurred below the laser scanning path. The overheating of CFRP led to significant decomposition and generated apparent porosity, where the relative height was negative. Therefore, zone C can be defined as the decomposition zone (DZ). At the edge of porosity, one can find a smooth surface with a dark color; such a phenomenon indicated that the plastic was carbonated, as shown in Figure 12(c-4). Indeed, the appearance of DZ reduced the effective joining zone between DP980 and CFRP, which led to the degradation of global joining strength.

### 4.5. Analysis of Interfacial Chemical Bonding

**Figure 13:**
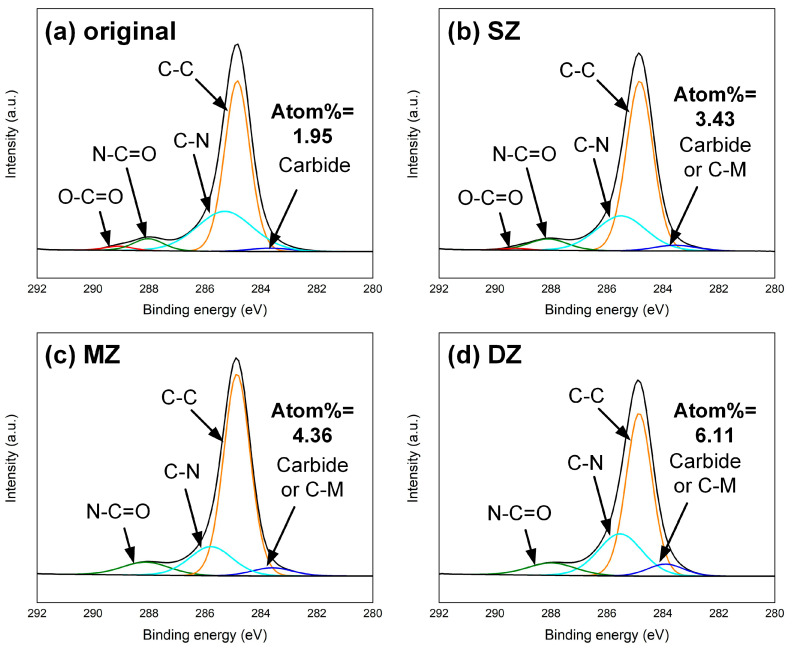
The high-resolution C1s XPS region spectra on the CFRP surface: (**a**) original surface, (**b**) SZ, (**c**) MZ and (**d**) DZ.

The XPS analysis was performed on each subregion of the fracture surface to clarify the interfacial bonding mechanism. The high-resolution C1s XPS region spectra on the CFRP surface were compared in Figure 13. The resin matrix of CFRP was PA6, whose chemical formula was [NH-(CH_2_)_5_-CO]_n_. The peaks located at about 284.85 eV, 285.3 eV and 288 eV represented C-C, C-N and N-C=O bonds [25,26,27], respectively. As the primary chemical bonds of the structural units of PA6, they can be detected in each subregion. The carboxyl group (-COOH) was the end of the PA6 molecule, which introduced the signal of the O-C=O bond at about 289 eV in the original CFRP surface. The peak at about 283.6 eV represented the residual carbide on cleaned CFRP [10], as shown in Figure 13a.

The hydrogen bonds of the metal oxide (or hydroxide) layer on the metal surface had the ability to react with the polar group, e.g., the carboxyl (-COOH) group in PA6 [28], which can provide effective joining force between the dissimilar materials. In SZ, the fluid plastics reacted with the metal surface insufficiently due to the limited interface temperature. Hence, the C-M bond was generated but there was still a weak signal of the O-C=O bond, as shown in Figure 13b. In comparison, the higher interface temperature of MZ was favorable for promoting the interfacial reaction and generating C-M bonds. Therefore, the signal of the O-C=O bond disappeared and the proportion of carbide or C-M increased to about 4.36 at.%. Moreover, the CFRP in the DZ was overheated significantly. The decomposition and even carbonization of plastic introduced much carbide here, which contributed to the strongest signal peak of carbide or C-M bond, as shown in Figure 13d.

**Figure 14:**
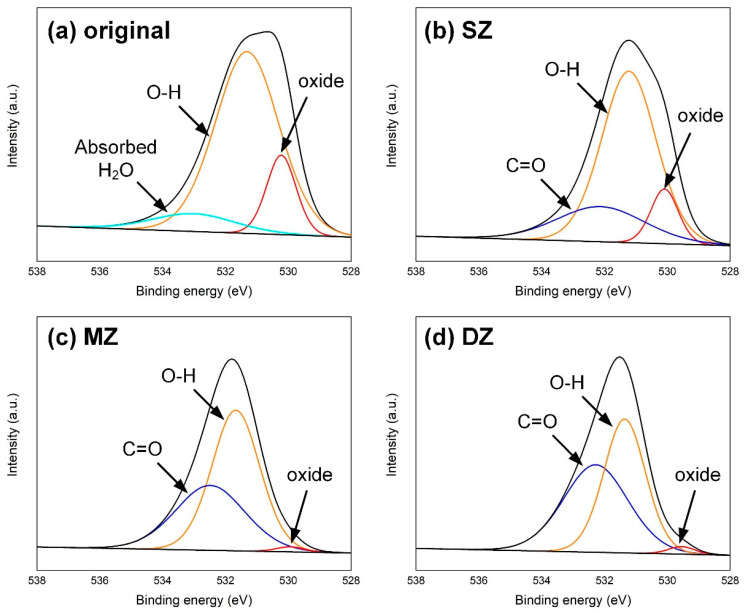
The high-resolution O1s XPS region spectra on the DP980 surface: (**a**) original surface, (**b**) SZ, (**c**) MZ and (**d**) DZ.

The high-resolution O1s XPS region spectra on the DP980 surface are given in Figure 14. The steel surface was polished and cleaned ultrasonically in ethanol solution. Therefore, the peaks located at about 530.4 eV, 531.5 eV and 533.2 eV represented the oxide of ferrite, hydroxide and absorbed H_2_O [29], respectively. After welding, the absorbed H_2_O disappeared due to the high temperature. A few plastics remained on the DP980 surface in the SZ; hence, one can detect the signal of the C=O bond at about 532.1 eV [30]. In the MZ, the better interfacial bonding resulted in more residual CFRP on the DP980 surface. Therefore, the intensity of the C=O bonds increased continuously, while the signal of oxide was covered, as shown in Figure 14c. Such a phenomenon was also observed in the DZ. The decomposition and carbonization of plastic led to much lamellar CFRP with a dark color on the DP980 sheet, which induced the largest intensity of C=O bonds.

### 4.6. Analysis of Interface Temperature Distribution

**Figure 15:**
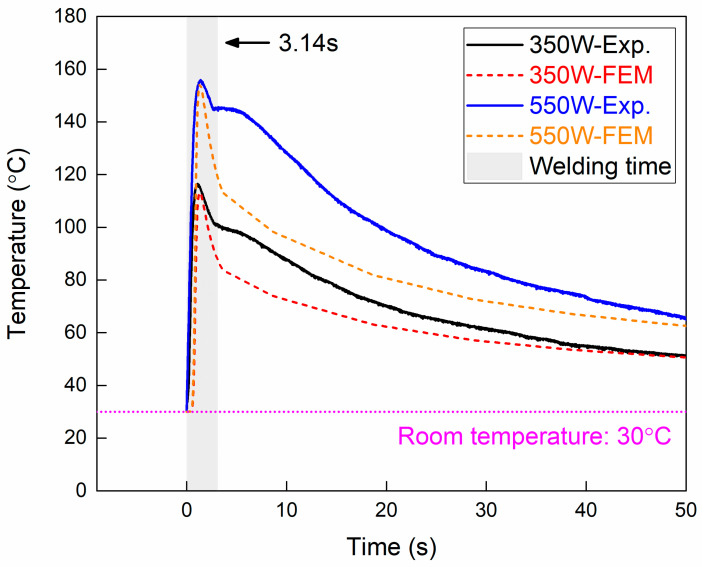
Comparison of measured thermal cycle with numerical prediction.

The interface temperature distribution of the DP980-CFRP LCW joint was analyzed numerically in the present research. Due to the excellent explicit scheme, the thermal process of LCW was finished within 51 s using an Intel Core i7-10700 CPU and 32 GB RAM on Windows 11 Pro system. Since the molten zone of CFRP was not as apparent as metals, the measured thermal cycle was employed to verify the effectiveness of the numerical model, as shown in Figure 15. The temperature increased rapidly when the laser source moved to the measured position. Considering the low thermal conductivity of DP980, the thermal cycle reached a peak at about 1.3 s. Then, the temperature decreased as the heat source moved away. However, the measured thermal cycles presented a clear inflection at the end of the welding time. The significant variation in cooling rate was induced by stopping the flow of Argon gas, whose cooling effect during welding was not considered in the numerical model. Generally, the discrepancy of peak temperature between measurement and prediction was within 3% (about 5 °C), which indicated that the developed numerical model was effective in predicting the heating process and peak temperature of LCW.

**Figure 16:**
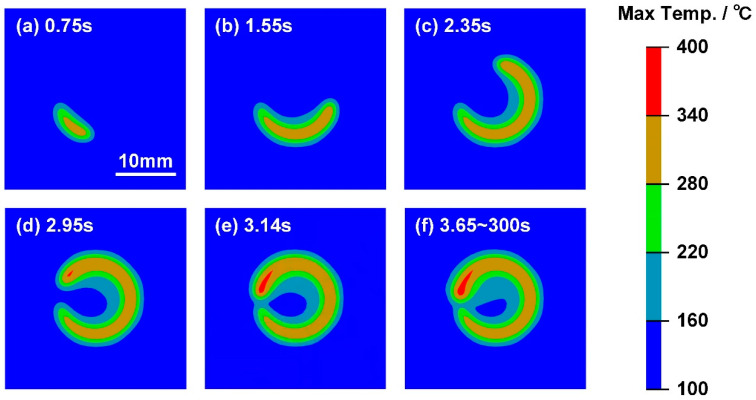
The variation in maximum interface temperature field in Case D: (**a**) 0.75 s, (**b**) 1.55 s, (**c**) 2.35 s, (**d**) 2.95 s, (**e**) 3.14 s and (**f**) 3.65–300 s.

The variation in maximum interface temperature field in Case D with 450 W laser power is shown in Figure 16. As depicted in Figure 16a–c, the CFRP was melted below the laser scanning path; hence, the molten zone increased with time. In ideal conditions, the average temperature of the welded joint increased during welding; therefore, the maximum interfacial temperature was consistently higher at the end of the scanning path. Therefore, one can find that the overheating of CFRP appeared at about 2.95 s, as shown in Figure 16d. In addition, the heating process was finished at 3.14 s, while the molten zone still grew larger until 3.65 s; such a phenomenon was driven by the heat flow from high-temperature DP980 to CFRP at the initial stage of cooling [21].

**Figure 17:**
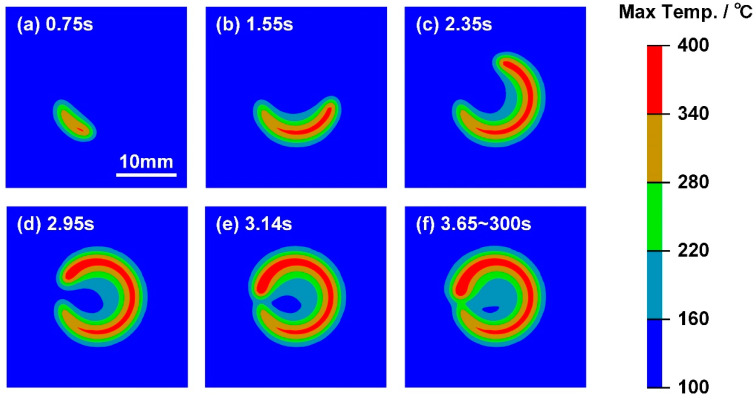
The variation in maximum interface temperature field in Case E: (**a**) 0.75 s, (**b**) 1.55 s, (**c**) 2.35 s, (**d**) 2.95 s, (**e**) 3.14 s and (**f**) 3.65–300 s.

The variation in maximum interface temperature field in Case E with 500 W laser power is shown in Figure 17. With a higher laser power and heat input, the maximum interface temperature will exceed the onset decomposition temperature of CFRP at the start of welding, as illustrated in Figure 17a. Therefore, the CFRP can experience a long high-temperature duration before significant decomposition. The generated porosity can reduce the effective bonding zone between dissimilar materials, which leads to the degradation of joining strength.

**Figure 18:**
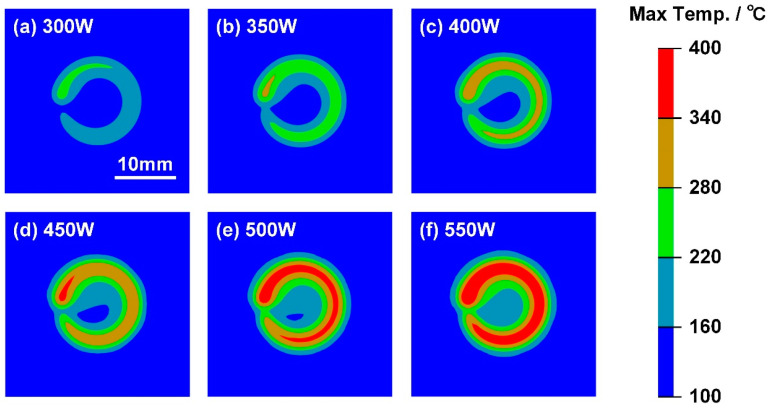
The maximum interfacial temperature distribution with different laser power: (**a**) 300 W, (**b**) 350 W, (**c**) 400 W, (**d**) 450 W, (**e**) 500 W and (**f**) 550 W.

The maximum temperature distribution on the DP980-CFRP interface with different welding conditions is compared in Figure 18. In Case A, the 300 W laser power was insufficient to melt much plastic. The MZ only appeared at the end of welding, which explained the natural fracture of the welded joint. When the laser power increased to 450 W, the MZ grew into a ring shape. Although the interface temperature exceeded 340 °C locally at the end of scanning, the duration was limited to induce a significant decomposition. Therefore, the joining strength reached a peak, as shown in Figure 18d. However, such a tendency was reversed in Case E and Case F. The overheating of CFRP led to a significant expansion of the DZ, which resulted in a smaller bonding area between dissimilar materials. The joining strength of LCW joints also decreased significantly.

**Figure 19:**
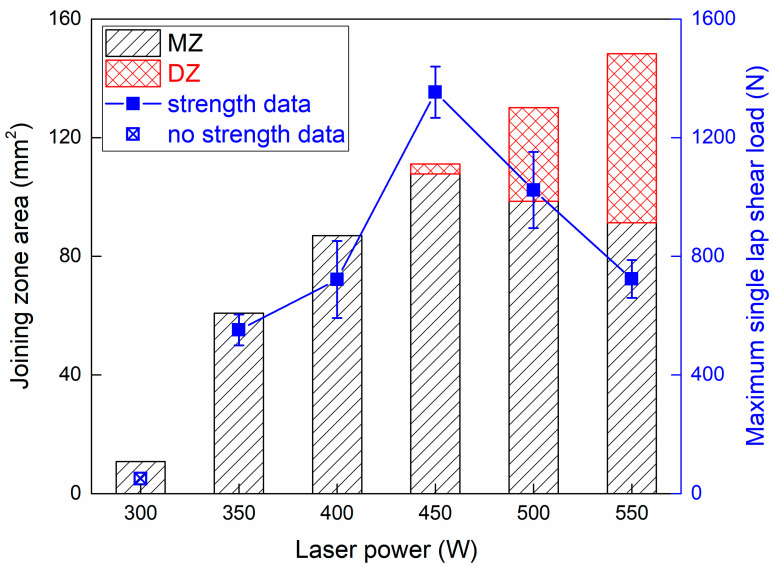
Relationship between joining zone area and joining strength.

Figure 19 compares the predicted joining zone area in the FE model to the joining strength. It should be mentioned that the SZ cannot be predicted by numerical analysis. With the continuously increased laser power and heat input, the joining zone expanded, while the area of the MZ and DZ was changed dynamically. Under 450 W, the expansion of the MZ improved the joining strength significantly. However, the overheating of CFRP led to the growth of DZ and reduction in MZ, which induced the degradation of joining strength. According to the simulation result of the joining zone area, the maximum joining strength in Case D can be calculated as 12.2 MPa, which is about 43% of the CFRP strength.

The interface temperature range in each subregion was different, which introduced distinct local bonding quality. Therefore, joining strength variation reflected the competitive result between joining zone expansion and local bonding quality change. Generally, the MZ played a more critical role than the DZ in strong bonding; the contribution of the SZ is also worth clarifying more deeply in further research.

## 5. Conclusions

In the present research, laser circle welding was employed to join DP980 and PA6-CFRP dissimilar materials. The influence of laser power on welding appearance, interfacial bonding quality and joining strength was analyzed experimentally. The subdivision of the joining zone was discussed based on the multi-scale characterizations of the fracture surface. The chemical bonding information in each subregion was clarified. A numerical model was developed based on the in-house code JWRIAN-Hybrid to predict the interface temperature distribution and discuss the variation in joining strength in detail. The following conclusions can be drawn:

1. Laser circle welding can join DP980 and PA6-CFRP successfully when the laser power was larger than 300 W with 12 mm/s scanning speed and 12 mm scanning diameter.
2. The LCW joints always presented a brittle fracture. The maximum joining strength reached about 1353.2 N (12.2 MPa), which was about 43% of the CFRP strength.
3. The joining zone of LCW joints can be subdivided into three parts according to the morphology of CFRP, which were defined as the squeezed zone (SZ), molten zone (MZ) and decomposition zone (DZ).
4. The chemical reaction between the O-C=O bond on the CFRP surface and the -OH bond on the DP980 sheet provided the joining force between dissimilar materials.
5. The influence of laser power on joining strength was dual character. The joining strength variation reflected the competitive result between joining zone expansion and local bonding quality change.

## Figures and Tables

**Table 1:** The chemical composition of DP980 (wt%).

C	Si	Mn	P	S	Cr	Ni	Mo	Al	Ti	Fe
0.09	0.56	2.63	0.013	0.009	0.17	0.014	0.023	0.025	0.02	Bal.

**Table 2:** Process parameters of laser circle welding between DP980 and CFRP.

Case	Laser Power (W)	Scanning Speed (mm/s)	Scanning Diameter (mm)	Specimen Number (n)
A	300	12	12	4
B	350	12	12	4
C	400	12	12	4
D	450	12	12	4
E	500	12	12	4
F	550	12	12	4

**Table 3:** Temperature-dependent thermal properties of CFRP.

**Temperature** **(°C)**	Specific Heat (J/(g⋅°C))	Thermal Conductivity (J/(mm⋅°C⋅s))	Density (g/cm^3^)
20.0	1.344	0.000427	1.1225
60.0	1.759	0.000435	-
160.0	2.231	0.000437	-
200.0	2.843	0.0004375	1.1225

**Table 4:** Temperature-dependent thermal properties of DP980.

**Temperature** **(°C)**	Specific Heat (J/(g⋅°C))	Thermal Conductivity (J/(mm⋅°C⋅s))	Density (g/cm^3^)
25.0	0.482	0.0325	7.66
200.0	0.556	0.0351	-
400.0	0.635	0.0342	-
600.0	0.785	0.0316	-
800.0	0.789	0.0297	7.66

## Data Availability

The data used to support the findings of this study are included within the article.
